# Sensory characteristics and consumer segmentation of fried sweetpotato for expanded markets in Africa

**DOI:** 10.1111/ijfs.14847

**Published:** 2020-11-05

**Authors:** Eric K. Dery, Edward E. Carey, Reuben T. Ssali, Jan W. Low, Suzanne D. Johanningsmeier, Ibok Oduro, Abena Boakye, Rachael M. Omodamiro, Hauwa L. Yusuf

**Affiliations:** ^1^ Department of Food Science and Technology Kwame Nkrumah University of Science and Technology UPO PMB Kumasi Ghana; ^2^ International Potato Center (CIP) Box 38785 Femusua, Kumasi Ghana; ^3^ International Potato Center (CIP) Box 25171 Nairobi 00603 Kenya; ^4^ Agricultural Research Service, Southeast Area Food Science and Market Quality & Handling Research Unit United States Department of Agriculture (USDA) Raleigh NC USA; ^5^ National Root Crops Research Institute P.M.B. 7006 Umuahia, Umudike 440001 Nigeria; ^6^ Bayero University P.M.B. 3011 Kano Nigeria

**Keywords:** Breeding, crispness, fries, *Ipomoea batatas*, profile, sweetpotato

## Abstract

Prepared foods are increasing in popularity in West Africa alongside rapid urbanisation. Growing demand for fried products calls for targeted breeding efforts to meet consumer needs, but little is known regarding consumer preferences. This research identified the sensory attributes of fried sweetpotato preferred by different consumer groups using a combination of consumer acceptance testing and descriptive sensory analysis. Market and community surveys identified three consumer segments in Ghana and Nigeria with contrasting preferences for fried sweetpotato sensory attributes. One group preferred crispy, crunchy, mealy and sweet fried sweetpotato; another preferred characteristic yam flavour and dry texture; and the third preferred uniform orange colour appearance, ripe plantain flavour and palm nutty flavour. Such consumer segmentation can help emerging West African fried sweetpotato industries identify target markets and provides valuable information to breeders, growers and retailers to prioritise attributes in their breeding, growing or product sourcing decisions.

## Graphical abstract

This research provides a guide to sweetpotato breeders for developing and deploying appropriate sweetpotato cultivars capable of meeting targeted consumer needs for sweetpotato fries and other fried sweetpotato products in distinct segments. It will also enable stakeholders in the sweetpotato value chain strategise in order to increase sweetpotato adoption.

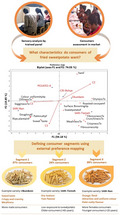

## Introduction

The use of sweetpotato as a food security crop in sub‐Saharan Africa (SSA) has long been recognised, but diversified utilisation of sweetpotato in the form of processed products is gradually being introduced in different countries and adopted. Sweetpotato is a nutritionally important crop and increased consumption has been attributed to several factors. Though primarily a starchy staple, it is also rich in antioxidants, vitamins and minerals in both its roots and leaves. Orange‐fleshed, provitamin A‐rich types have been well‐established for their ability to combat vitamin A deficiency, when combined with a nutrition education component (Low *et al*., 2007; Hotz *et al*., 2012; Tomlins *et al*., 2012; Truong et al., 2018). Sweetpotato contributes greatly to the fresh market industry in Nigeria, which serves street food vendors and other formal and informal markets (Onumah *et al*., 2012). Despite its versatility and increased popularity in SSA, consumption of sweetpotato in Ghana has been limited due to culinary, nutritional, sensory and lifestyle patterns. Consumers cite sweet taste and textural properties as the most important factors affecting consumption (Sam & Dapaah, 2009; Adu‐Kwarteng *et al*., 2014). Breeders and product developers are, therefore, exploring various options to overcome barriers to increased consumption.

Traditionally, sweetpotato is prepared boiled, roasted, fried or pounded and can be used in diverse local food recipes or baked products (Abidin *et al*., 2015). In West Africa, sweetpotato is perceived as a snack food crop (Sugri *et al*., 2012). However, the potential of sweetpotato as a snack food in Ghana and Nigeria has not been harnessed in the formal market space. In the informal sector, chunk sweetpotatoes are sold in sweetpotato producing regions of Ghana, as described by Ssali *et al*. (2020). Snack foods are handy, convenient and usually eaten between main meals. With increased urbanisation and fast‐lane lifestyles, snack food consumption has increased (Staatz and Hollinger, 2016). Though there are many forms of snack food, fried products are common snacks, accessible by all income groups. Frying involves the immersion of food in heated edible oil/fat causing moisture in the product to evaporate as vapour, leaving spaces to be occupied by oil (Varela *et al*., 2008; Tortoe *et al*., 2014; Fetuga *et al*., 2014; Sato *et al*., 2018). This complex interplay of moisture, vapour and oil with the help of a heating medium changes the physical and chemical properties of the products, which affects consumer acceptability. Attributes generally desired by consumers, such as crispness, crunchiness, brown tint colour and fried flavour, has been found to be a function of its starch and sugar content of the sweetpotato (Varela *et al*., 2008; Caetano *et al*., 2018; Sato *et al*., 2018; Laryea *et al*., 2018). Sato *et al*. (2018) reported that high dry matter roots generally produce French fries with firm and dry textures while low dry matter was related to moist and smooth inner textures. In their work, higher sugar content in finished fries was associated with increased perception of moistness, softness and cohesiveness of the inner portion of the fry. Reports by Laryea *et al*. (2018), Caetano *et al*. (2018) and Tortoe *et al*. (2014) also suggest a development of brown colour due to reducing sugars undergoing the Maillard reaction. However, Sato *et al*. (2018) and Laryea *et al*. (2018) did not perform consumer sensory evaluations to determine which of these sensory properties drive consumer preference.

Traditional methods of sensory evaluation regress average hedonic ratings onto mean analytical data with the assumption that consumers exhibit similar behavioural patterns, thereby representing all consumers with a single mean value (Stone & Sidel, 2004; Guinard, 2002; Lawless & Heymann, 1999). However, the heterogeneity of consumers in preferences makes other sensory analysis techniques such as internal and external preference mapping relevant in identifying different consumer segments and drivers of liking (Guinard, 2002; Lawless & Heymann, 1999). While internal preference mapping analyses hedonic ratings of consumers for a product set using the covariance matrix with summary preference direction, external preference mapping uses a number of regression models (linear to quadratic) to regress preferences of each consumer onto the first two principal components of product sensory characteristics derived from descriptive sensory analysis or instrumental measurements. These models have been employed by several authors to identify consumer segments in various products (Leksrisompong *et al*., 2012; Symoneaux *et al*., 2012; Amyotte *et al*., 2017; Bowen *et al*., 2019; Sharma *et al*., 2020). Identification of different consumer groups enables expansion of markets for new or improved products developed through targeted breeding and informs policy development such as health interventions, promotion and awareness strategies. Recent research has, therefore, found sensory evaluation to be critical in product profiling. In Ghana, this in‐depth approach has been generally lacking in plant breeding programmes, resulting in several released varieties that were not well adopted by consumers. Promotion and dissemination of sweetpotatoes have been increasingly challenged by the lack of information about different consumer groups and their preferred product attributes. Therefore, this research aims to clarify consumer preference for sweetpotato fries made from different cultivars in Ghana and Nigeria, and relate this to sensory attributes that drive preferences of distinct consumer groups for fried products.

## Materials and method

### Sweetpotato samples

**Figure 1:**
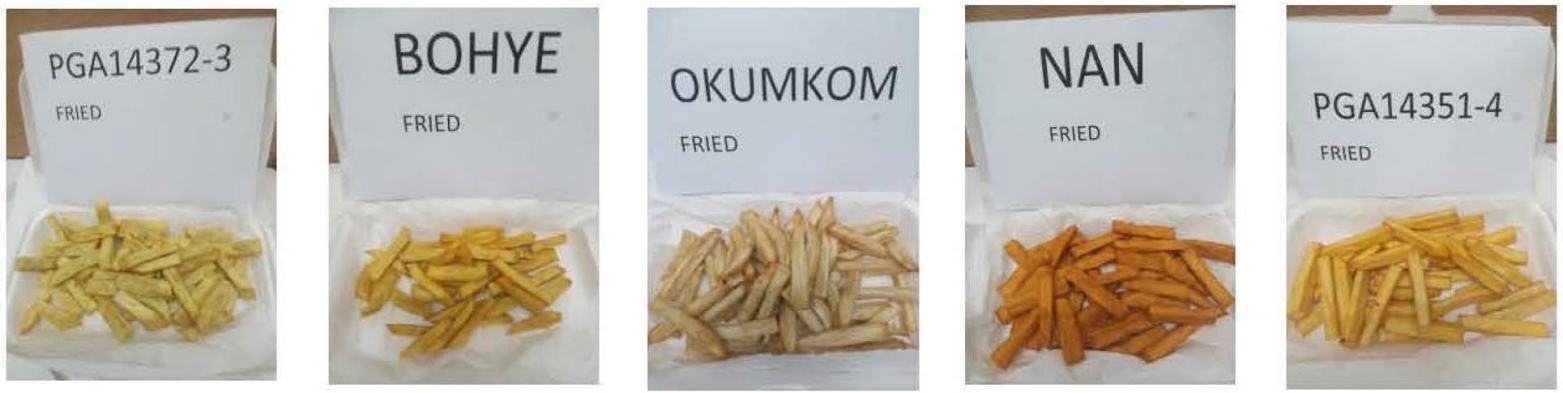
Pictures of fried sweetpotatoes used for DSA and Ghana market survey (NB: PGA14372‐3 was recently released as SARI‐Tiemeh).

Consumer and descriptive sensory analysis was conducted using two sets of sweetpotato varieties that were evaluated in market or community settings (termed market or community surveys). The market survey set included five contrasting sweetpotato cultivars from breeding trials of the Crop Research Institute and Savanna Research Institute of the Council for Scientific and Industrial Research, Ghana and the International Potato Center. These included both released cultivars, and clones at the advanced stage of the varietal selection process (Table 1; Fig. 1). Another set of seventeen cultivars was used in the community‐based survey in Ghana and Nigeria to further identify differences in consumer preferences (Table 1). These cultivars were proposed by participants of focus group discussions in communities during a gendered mapping survey of local fried sweetpotato products (Forsythe *et al*, 2018). Cultivars were harvested and kept in a cool dry place for 3 days before each survey.

**Table 1:** Sweetpotato cultivars used for market or community surveys in Ghana and Nigeria

Varieties	Status	Country of origin	Flesh Colour	Study	Breeder/FGD inference
SARI‐Nan	Released	Ghana	Deep Orange	Market survey	Sweet type
PGA14351‐4	Advanced selection	Ghana	Pale Orange	Market survey	Low sweet type
CRI‐Bohye	Released	Ghana	Pale Orange	Market survey	Low sweet type
Okumkom	Released	Ghana	Light Cream	Market survey	Sweet type
SARI‐Tiemeh	Released	Ghana	Light Yellow	Market survey	Low sweet type
Kuffour	Farmers variety	Ghana	Deep Orange	Community survey	OFSP type
Obare	Released	Ghana	Pale Cream	Community survey	Most preferred
Amuskwera	Farmer variety	Ghana	Deep Cream	Community survey	Least preferred
Purupuru	Farmer variety	Ghana	Deep Cream	Community survey	Moderately preferred
Dan Barmawa	Farmer variety	Nigeria	Deep Cream	Community survey	Moderately preferred
Aragbe	Farmer variety	Nigeria	Light Yellow	Community survey	Least preferred
Alausa	Famer variety	Nigeria	Light Yellow	Community survey	Most preferred
Elege	Farmer variety	Nigeria	Light Yellow	Community survey	Least preferred
Madagali	Farmer variety	Nigeria	Light Yellow	Community survey	Moderately preferred
Dan Silver	Farmer Variety	Nigeria	White	Community survey	Least preferred
Tomude	Farmer variety	Nigeria	Light Yellow	Community survey	Moderately preferred
Pakurumon	Farmers variety	Nigeria	Light Yellow	Community survey	Moderately preferred
Dan Bakalori	Farmer variety	Nigeria	Light Cream	Community survey	Most preferred
Mother's Delight	Released	Nigeria	Deep orange	Community survey	OFSP type
Dan Izala	Farmers variety	Nigeria	Deep Cream	Community survey	Most preferred
Dan China	Farmers variety	Nigeria	Light Yellow	Community survey	Most preferred

Materials used for the market survey were described by the breeding programme as either sweet or low sweet types. While materials used for the community survey were rated for suitability for frying by focused group discussions (FGDs) as either most preferred, moderately preferred or least preferred. Colour measurement was by visual observation as employed by CIP‐Ghana.

### Fried sweetpotato sample preparation

Samples for the consumer tests in the market survey and for descriptive sensory analysis were prepared by cutting peeled, fresh roots manually into average dimensions of 0.7 cm × 0.7 cm × 7.0 cm with a stainless‐steel knife. The ends of strips with tapered edges were cut off before frying. Each frying batch contained about 20 strips of sweetpotato in 120 ml oil and oil was changed after frying each genotype. Deep fat frying was done using a stainless‐steel pan containing a refined palm olein (Frytol oil brand). Oil was heated to a temperature of 180°C before frying for about 8–10 min. Samples were placed on a white paper towel inside a clean disposable bowl to drain excess oil for about 2 min before wrapping them with aluminium foil. Sensory evaluation was conducted within 10 mins after frying with initial inner strip temperature of about 80°C. Samples for the consumer sensory tests in the community survey were prepared by expert fryers at each community using local common practices that varied slightly as described by Ssali *et al*. (2020). The major difference with normal practice is that the slices for the market survey were prepared as ‘French fries’, not the traditional chunk fries.

### Descriptive sensory analysis (DSA) (Ghana only)

**Figure 2:**
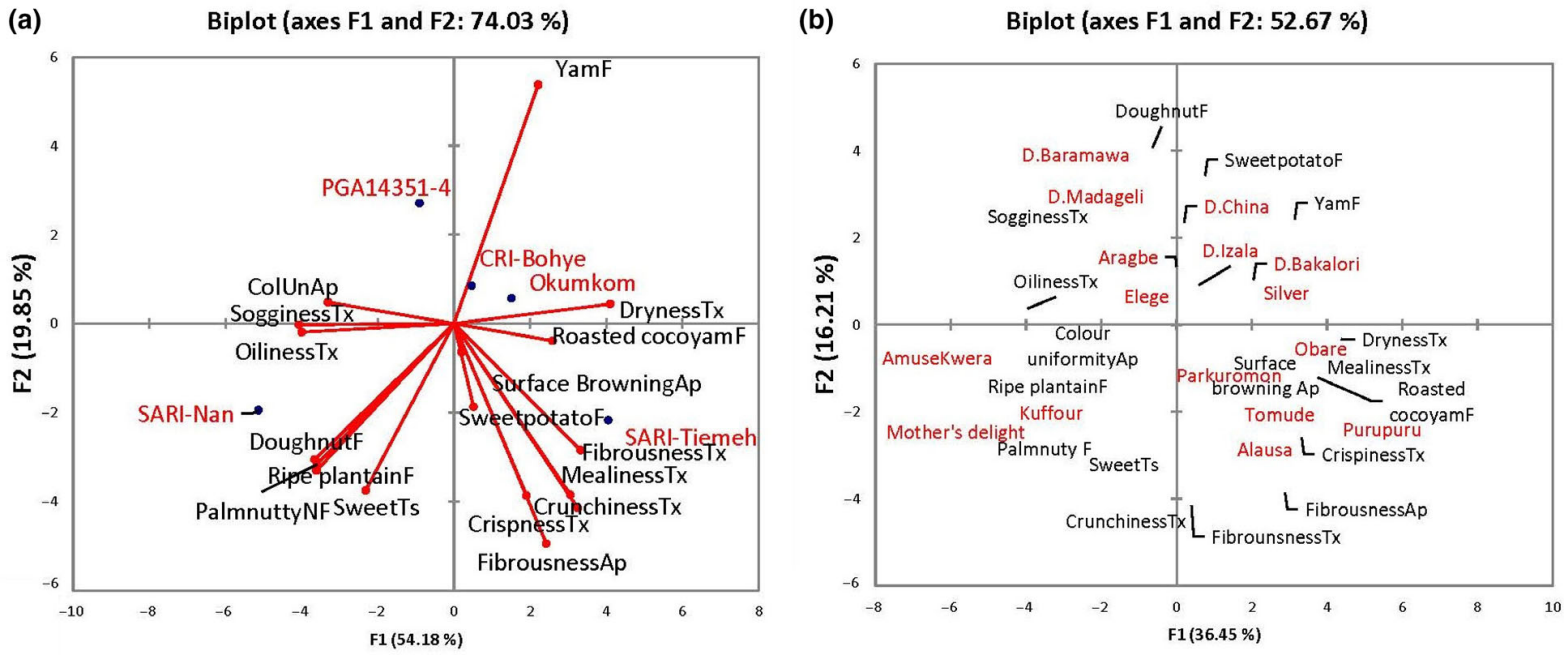
Principle component analysis with (A = 74.03 % and B = 52.67 %) variability of all descriptive terms explained for all cultivars for (a) Cultivars used for the market survey in Ghana only, and (b) Cultivars used for community survey in Ghana and Nigeria (Cultivar names in red; Characteristics ending in Ap, Tx and F represent appearance, texture and flavour, respectively).

Eight panellists were used to profile the two sets of sweetpotato materials (Table 1) using a lexicon generated by the panel (Table 2 and Fig. 2). The ages of the panellists ranged from 25–35 years and consisted of three females and five males. Panellists were selected based on their performance in the lexicon generation phase (Dery, personal communication). Panel candidates received a total of 50 h of training on scaling, selection of reference materials and definition of terminologies before sample evaluation. Training was carried out by asking panellists to propose descriptors and corresponding reference materials to describe the different attributes as perceived by the sense of vision, smell, taste and touch whiles evaluating a set of different materials. The number of descriptors was then reduced through group discussions and consensus to include only the critical descriptors capable of describing fried sweetpotatoes. Panellists were also made to score different samples with different scales to determine which scale was suitable. Suitability of scale was determined by monitoring panel performance using the various scales. Prior to each test evaluation, panellists observed and tasted reference materials to refresh their memories. In the test evaluation, five different genotypes were evaluated using a completely randomised design for three days and three repetitions in sensory booths. Data were first collected on a sheet of paper before transferring to computer for analysis. All five cultivars were evaluated each day with a repeated evaluation on subsequent days in the morning (10 am‐12 pm). A three‐digit code, generated using statistical software XLSTAT (Version 2014.5.03; Addinsoft 1995–2014), was randomly assigned to each sample before serving monadically at a starting inner temperature of 75°C–80°C. A 0–9 discrete scale with 0 representing the absence of an attribute and 9 its highest intensity (Bugaud *et al*., 2011) was used by the panel to scale the intensity of product attributes. A total of 17 attributes including appearance, texture, flavour and taste attributes were evaluated due to their perceived relevance by panellists. Distilled water and unsalted crackers were used as palate cleansers between samples.

**Table 2:** Definition of sensory attributes of fried sweetpotatoes used by trained panel with a scoring system of 0 representing absence of the attribute to 9, the highest intensity

Attribute class	Standard Terminology	Definition	Reference
Appearance	Colour Uniformity	The degree of uniformity of the dominant natural colour	Yam (*Dioscorea rotundata*)
	Surface Browning	The degree of browning or brown spots due to frying on a cross section	Brown biscuit (Cream crackers)
	Fibrousness	The degree of threadlike appearance on the surface of a cross section	Cassava
Texture	Crispness	The snappy sound heard when breaking a fry	Yam
	Sogginess	Impression of the degree of oil or moisture absorbed by the product when pressed between fingers	Fried ripe plantain
	Oiliness	Oil on the surface of the product when swiping the surface with the fingers	Yam
	Fibrousness	The degree of threadlike fibre on the tongue after chewing	Cassava
	Crunchiness	Sound heard during the first few chews	Cucumber
	Dryness	The degree of dryness of the fries during chewing	Yam
	Mealiness	The degree to which fries feel mealy (‘foodie’ or ‘floury’) in the mouth	Yam
Flavor	Sweetpotato‐like	Flavour associated with freshly fried sweetpotatoes	Sweetpotato (cultivar *Ogyefo*)
	Yam‐like	Flavour associated with freshly fried pona yam	Yam
	Roasted Cocoyam	Flavour associated with freshly roasted cocoyam	Cocoyam
	Ripe Plantain	Flavour associated with fried ripe plantain	Fried ripe plantain
	Palm nutty	Flavour associated with freshly boiled palm nut	Palm nut
	Doughnut (‘Bofrot’)	Flavour associated with fried fermented bread flour	Doughnut (‘Bofrot’)
Basic taste	Sweet	Basic taste stimulated by sugar	White table sugar
	Bitter	Basic taste associated with caffeine	Freshly brewed coffee
	Salty	Basic taste associated with sodium chloride	White table salt
	Bitter Aftertaste	Bitter taste perceived after swallowing which was not initially perceived during chewing	Freshly brewed coffee

### Consumer acceptance tests

#### Market survey (Ghana only)

Consumer preference tests of the five varieties in the market survey were carried out in four major Ghanaian regional markets purposively selected. Three of these regions (Upper East, Volta and Central) are known to have major sweetpotato producing communities. Bawku in the Upper East Region, Cape Coast in the Central Region and Akatsi in the Volta Region were selected for being the major regional markets and Agbogbloshie, Malata, Kaneshie and Accra Mall markets in the nation’s capital, Accra, were selected due to high levels of economic activities in those markets.

Sweetpotato fries were prepared and served to randomly selected consumers at the selected markets. In total, 332 consumers evaluated samples across the four regions. Samples were prepared and coded with three letters generated using XLSTAT software (Version 2014.5.03; Addinsoft 1995‐2014). Consumers were asked to evaluate fries and rate them for overall liking, using a 9‐point hedonic scale (1 = Extremely dislike through 9 = Extremely Like). Samples were served on white disposable plates with subjects handling samples with disposable white tissue papers. Consumers were asked to rinse their mouths with water between different samples.

#### Community survey (Ghana and Nigeria)

Following the gendered mapping for fried sweetpotato product in Kano and Kwara States in Nigeria and Bawku in the Upper East Region of Ghana, consumer preference tests were conducted in the communities with 191 respondents (90 males (M), 101 females (F)) (Ssali *et al*., 2020). Sweetpotato cultivars used had been identified by the communities as most preferred, moderately preferred or less preferred; also a dominant orange‐fleshed sweetpotato cultivar in the region was used (Table 1). Conducive testing area conditions were created for each panel; this consisted of a separate table with chairs for the enumerator, a translator and one panellist at a time there were about four of these at a time in each testing site. Selected panellists waiting for their turn to evaluate the product, were comfortably seated a good distance from the main testing area. A separate preparation area was provided for expert fryers recruited for the sample preparation. A bottle of water and slices of cucumber (a commonly consumed fruit in the locality with ability to remove mouth coating from fatty foods) were used as palate cleansers. Each panellist was first asked to rinse the mouth with water prior to the assessment of the first product and then directed to cleanse the palate with a slice of cucumber which was then washed down with water in between tasting of the products The samples were served in sequential monadic fashion. Each panellist was made to evaluate all four samples using liking [on a 9‐point hedonic scale ‐ 1 (extremely dislike), 5 (neither like nor dislike), 9 (extremely like)].

### Statistical analysis

#### DSA

Data were subjected to one‐way analysis of variance (ANOVA) with means separation conducted using Tukey test at 5% significant level for each sensory attribute (JMP Pro 11.0.0, 2013, SAS Institute, Inc). Principal component (PCA) analysis using the correlation matrix of significantly different sample attributes was used to visualise how sweetpotato cultivars were differentiated across sensory attributes.

#### Consumer preference analysis

Consumer liking scores in both surveys were subjected to one‐way ANOVA using JMP Pro 11 and mean separation performed by Tukey test. Cluster analysis was also carried out using Agglomerative Hierarchical Clustering (AHC) to group consumers into different segments based on shared characteristics using XLSTAT (Version 2014.5.03; Addinsoft 1995‐2014). One‐way ANOVA was then performed on the overall liking scores for each cluster using JMP Pro 11 and mean separation by Tukey test. An external preference mapping (PREFMAP) was created using a vector model by regressing consumer cluster groups onto the factor scores of the first two principal components from the DSA.

## Results and discussion

### Diversity in sensory attributes of sweetpotato cultivars by trained panellists

#### Appearance

The appearance of fried sweetpotato was measured by uniform colour, surface browning and fibrousness (Table 3 and Supplementary Table 1). Most of the varieties in the study were rated highly for colour uniformity, with CRI‐Bohye having the lowest intensity (5.3) in market survey varieties (Table 3) and Obare (6.4) in the community survey varieties (Supplementary Table 1). Darkening due to processing, browning due to Maillard reaction, fibre, uneven distribution of oil and water after frying were noted by the panel as some of the factors contributing to distortion of natural colours. Deep orange‐fleshed varieties (SARI‐Nan, Kuffour, Mother’s delight) had high colour uniformity but lower surface browning compared to lighter fleshed varieties. This could be attributed to the masking effect of pigments producing these colours. Carotenoids are the main pigmented compounds found in yellow‐ and orange‐fleshed varieties (Tomlins *et al*., 2012; Truong et al., 2018). Generally, surface browning was low in all varieties (1.4‐3.4 in market survey varieties and 0.2–4.7 in community survey varieties), suggesting that frying was carefully carried out. However, differences surface browning could be attributed to possible reducing sugar contents in the different varieties (Caetano *et al*., 2018; Laryea *et al*., 2018).

**Table 3:** Average scores of descriptive sensory characteristics for five French fried sweetpotato cultivars used for Ghanaian Market Survey

Descriptors	Cultivar				
	Bohye	Nan	Okumkom	PG14351‐4	SARI‐Tiemeh
Colour Uniformity (Ap)	5.3 ± 0.40^b^	7.8 ± 0.57^a^	5.4 ± 0.45^b^	7.5 ± 0.32^a^	5.6 ± 0.19^b^
Surface browning (Ap)	3.4 ± 1.19^a^	2.5 ± 1.56^ab^	3.3 ± 0.56^a^	1.4 ± 0.19^c^	2.0 ± 0.65^bc^
Fibrousness (Ap)	1.8 ± 1.00^a^	1.5 ± 0.26^ab^	1.6 ± 0.25^ab^	1.0 ± 0.38^b^	2.3 ± 0.66^a^
Crispiness (Tx)	1.4 ± 1.26^bc^	1.0 ± 0.19^cd^	2.2 ± 0.80^b^	0.5 ± 0.26^d^	3.5 ± 0.63^a^
Sogginess (Tx)	3.4 ± 0.33^b^	5.0 ± 0.13^a^	2.6 ± 0.07^c^	3.3 ± 0.52^bc^	1.6 ± 0.57^d^
Oiliness (Tx)	2.8 ± 0.55^b^	4.5 ± 0.14^a^	3.0 ± 0.56^b^	3.1 ± 0.43^b^	1.8 ± 0.44^c^
Fibrousness (Tx)	1.3 ± 0.63^ab^	0.7 ± 0.07^b^	1.0 ± 0.29^ab^	0.6 ± 0.13^b^	1.6 ± 0.75^a^
Dryness (Tx)	4.5 ± 0.65^bc^	2.0 ± 0.44^d^	5.4 ± 0.33^ab^	4.1 ± 0.44^c^	6.0 ± 0.19^a^
Crunchiness (Tx)	0.9 ± 0.45^c^	1.0 ± 0.07^bc^	1.7 ± 0.26^b^	1.0 ± 0.38^bc^	3.3 ± 0.45^a^
Mealiness (Tx)	5.9 ± 0.32^b^	6.0 ± 0.25^b^	5.6 ± 0.38^b^	6.0 ± 0.75^b^	6.9 ± 0.33^a^
Sweetpotato (F)	5.8 ± 0.25^b^	6.4 ± 0.50^ab^	6.9 ± 0.22^a^	6.1 ± 0.45^ab^	6.4 ± 0.13^ab^
Yam (F)	3.7 ± 0.52^b^	0.8 ± 0.40^d^	5.3 ± 0.51^a^	5.0 ± 0.13^a^	3.0 ± 0.32^c^
Roasted cocoyam (F)	0.5 ± 0.44^b^	0.6 ± 0.14^b^	1.5 ± 0.38^a^	0.8 ± 0.07^b^	1.2 ± 0.62^ab^
Ripe plantain (F)	1.0 ± 0.65^b^	4.3 ± 0.33^a^	0.6 ± 0.25^b^	0.7 ± 0.38^b^	0.5 ± 0.13^b^
Palm nutty (F)	1.5 ± 0.36^b^	5.5 ± 0.26^a^	0.3 ± 0.19^c^	0.5 ± 0.07^c^	0.3 ± 0.00^c^
Doughnut (F)	1.3 ± 0.41^b^	4.0 ± 0.25^a^	1.4 ± 0.29^b^	1.7 ± 0.63^b^	1.3 ± 0.51^b^
Sweet (T)	2.5 ± 0.22^c^	6.7 ± 0.40^a^	5.3 ± 0.13^b^	2.5 ± 0.26^c^	3.3 ± 1.26^c^

Descriptors ending: Ap = Appearance, Tx = Texture, F = Flavour, T = Taste.

Mean values in rows with different superscript letters indicate significantly different results at *P* < 0.05. Intensities were generated using 0‐ to 9‐ scale with 0 signifying absence of a descriptor and 9 being highest intensity of a descriptor.

#### Texture

Textural attributes (Table 3 and Supplementary Table 1) are very critical to acceptability of fried products globally. Crispness and/or crunchiness have frequently been cited as the most important criteria for determining acceptability (Varela *et al*., 2008; Tortoe *et al*., 2014). Generally, textural attributes are a function of starch composition of the product as well as processing factors such as time of frying, temperature of frying oil, type of oil and size of fries (Varela *et al*., 2008, Fetuga *et al*., 2014, Caetano *et al*., 2018, Sato *et al*., 2018). Sweetpotato cultivars with high dry matter generally produce fries with high crispy texture. In the first set of varieties (market survey varieties), SARI‐Tiemeh (30.8% dry matter content) and Okumkum (32.8% dry matter content) were described by the panel as having the highest crispness, crunchiness and dryness, and hence stood a greater chance of consumer acceptance. Comparatively, fries have lower crispness compared to thinly sliced chips because of crust formation (Varela *et al*., 2008; Fetuga *et al*., 2014) and this could be the reason for the generally low crispness values among the five cultivars, ranging from 0.5 to 3.5. Dan Silver, Obare, Purupuru, Dan Bakalori were also described by panel to have high crispy texture among the community survey varieties. The deep orange‐fleshed cultivar, SARI‐Nan (28.4% dry matter content), was observed to have the highest soggy and oily attributes, in line with previous reports (Tomlins *et al*., 2012; Laryea *et al*., 2018). Orange‐fleshed cultivars utilised to date in West Africa have typically had lower dry matter content than white‐fleshed local varieties, and the complex interaction between moisture, oil and heat transfer during frying could have contributed to the soggy and oily nature of SARI‐Nan (Varela *et al*., 2008; Fetuga *et al*., 2014; Sato *et al*., 2018). Understandably, Amuskwera, Mother’s Delight, Madagali, Dan China, Aragbe, Elege and Kuffour had high oiliness (Supplementary Table 1) and could be the reason for them being preferred least by fryers (Table 1) (Ssali *et al*., 2020).

#### Flavour

Flavours are generally difficult to describe due to low levels of flavour compounds and the challenge of distinguishing multiple flavour attributes at one time. All of the cultivars had high levels of characteristic sweetpotato flavour. With the exception of characteristic sweetpotato and yam flavours, most of the other flavours such as palm nutty, ripe plantain and doughnut flavours were barely perceived in white/cream‐fleshed varieties. However, the deep orange‐fleshed cultivar (SARI‐Nan) (Table 3) and Kuffour and Mother’s delight (Supplementary Table 1) had highly characteristic flavours of ripe plantain, palm nutty and doughnut‐like. These flavours might be related to beta‐carotene compounds and sugars since they were particularly noticed in orange‐fleshed cultivars, or could also be related to the high oil absorption of this cultivar. The intensity of orange colour in sweetpotatoes has been found to be closely related to the amount of beta‐carotene present (Tomlins *et al*., 2007; Tomlins *et al*., 2012). Palm fruits have also been cited as one of the major plant sources of beta‐carotene (Santos *et al*., 2015) and could perhaps explain the close association of this flavour in orange‐fleshed cultivars. In other studies, pumpkin, carrot and apricot flavours were associated with orange‐fleshed cultivars (Leksrisompong *et al*., 2012; Tomlins *et al*., 2012). Yam flavour was highest in high dry matter cultivars, especially Okumkom (Table 3) and Obare, Purupuru, Aragbe, Alausa, Dan Bakalori and Dan China (Table S1). In Ghana, white yam (*Dioscorea rotundata*), popularly called Pona, is the most widely consumed and preferred root and tuber variety (Tortoe *et al*., 2014) and its characteristic flavour is a strong driver of consumer preference.

#### Taste

Due to the overlapping usage of flavour and taste attributes in sensory descriptions, taste attributes were described as perceived by only the tongue which included the four basic taste sensations (sweet, sour, bitter, salt) and umami while flavour attributes were described to include aroma through the nasal cavity as well as through the mouth. Sweetpotato is generally a sweet crop due to its ability to easily breakdown starch to maltose as a result of amylase activity and to the presence of other sugars (Dziedoave et al., 2010; Owusu‐Mensah *et al*., 2016; Adu‐Kwarteng *et al*., 2014; Kays *et al*., 2005; Kitahara et al., 2017). Therefore, sweet taste was the predominant taste attribute perceived by the panel. SARI‐Nan was found to be the sweetest (6.7) with PGA14351‐4 and CRI‐Bohye were perceived as barely sweet (2.5) (Table 3). For the second set of varieties, Amuskwera (5.7) was the sweetest in Ghana and Dan Bakalori (4.5) in Nigeria (Table S1). This suggests that, flesh colour does not correlate with sweet taste even though most orange‐fleshed varieties are highly sweet. Other taste attributes, including sour and bitter tastes, were barely perceived and significant differences were not observed among cultivars, even when other taste attributes were present.

Generally, Okumkom, CRI‐Bohye and SARI‐Tiemeh were similar in their overall sensory attribute profiles and were characterised mainly by textural attributes such as crispness, crunchiness, mealiness, dryness and fibrousness, as well as roasted cocoyam and sweetpotato flavours. SARI‐Nan was characterised predominantly by flavour and sweet taste attributes together with oily and soggy textures. Similar attributes to SARI‐Nan were found with orange‐fleshed varieties Mother’s Delight (Nigeria) and Kuffour (Ghana) used for the community surveys (Fig. 2b). Obare, Pakurumon, Dan China, Dan Izala, Dan Bakalori, Dan Silver, Tomude, Alausa and Purupuru were characterised by surface browning and fibrous appearance, crispy, mealy and dry textures, roasted cocoyam and yam flavours. These varieties seem to have characteristic attributes suitable for frying and are similar to Okumkom and SARI‐Tiemeh used in the Ghana market survey.

### Consumer preferred cultivars and segmentation

**Figure 3:**
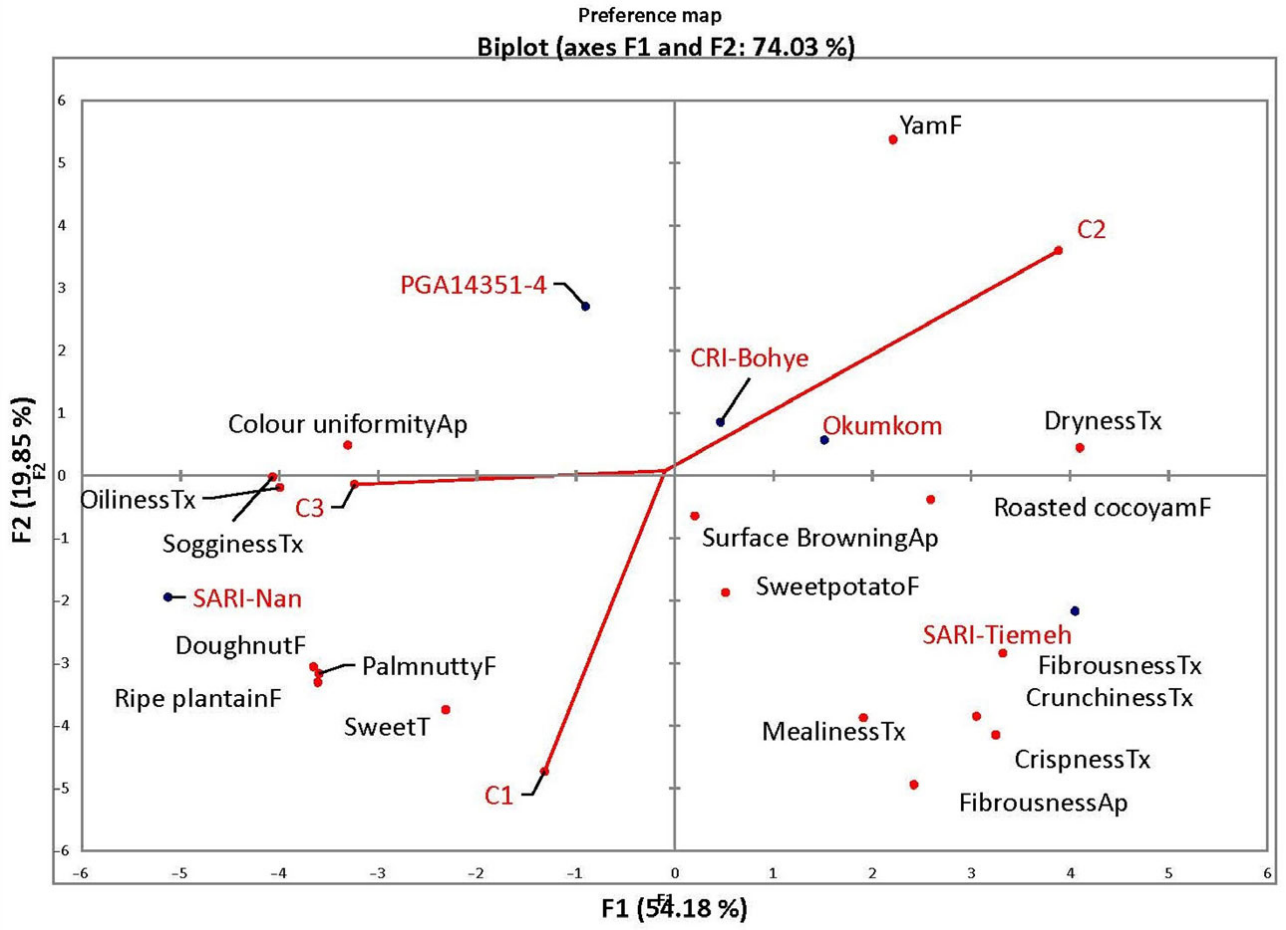
Consumer segmentation using external preference mapping on clusters derived from consumer preference tests of sweetpotato samples from market surveys in Ghana (C1 = Cluster1, C2 = Cluster2, C3 = Cluster3; Cultivar names in red; Characteristics ending in Ap, Tx and F represent appearance, texture and flavour, respectively).

Fried products generally appeal to consumers in a special way due to enhanced flavours, sweetness and appearance. Therefore, all cultivars evaluated in both surveys met the threshold of minimal acceptability (overall acceptability> 5 on the hedonic rating scale) across the locations), regardless of the different sensory attributes (Table 4A and B; Fig. 3 for Ghana market survey). The deep orange‐fleshed variety (SARI‐Nan) was consistently ranked highest in the various Ghanaian markets (Table 4A). This is consistent with findings in US consumers that rated the deep orange‐fleshed cultivar, ‘Evangeline’ as more well liked for colour than the common orange‐fleshed, ‘Covington’ (Barkley *et al*., 2017). The overall preference for the deep orange‐fleshed cultivar (SARI‐Nan) could also be due to other extrinsic factors such as knowledge about its nutritional value, processing and culinary properties, and other factors (Tuorila, 2015; Ssali *et al*., 2020). However, when using farmer varieties proposed by FDGs in the community survey, Obare (cream‐fleshed) was the highest preferred variety in Ghana even though it was not significantly different from the deep orange‐fleshed Kuffour and cream‐fleshed Amuskwera. This is in line with the findings of Sugri *et al*. (2012), where no significant difference was observed between Obare and Kuffour. In Nigeria, Mother’s Delight was the least preferred variety compared to other farmer varieties even though it was generally considered acceptable (Table 4B). In addition to the inherent properties of the farmer varieties, familiarity of the varieties could have also played an important role in preference. Obare is the predominant and widely consumed variety in Bawku (Ghana) and is sometimes exported to neighbouring country (Burkina Faso) (Sugri *et al*., 2012). The least sweet cultivar (PGA14351‐4) also ranked high (second to only SARI‐Nan) in southern Ghana (Table 4A), where yam is the preferred staple over sweetpotato even though both crops are consumed in these locations. In contrast, it ranked lowest in Bawku, the Northern part of Ghana, where sweetpotato is a major staple. This could be as a result of neophobia^1^ (Tuorila, 2015) since sweetpotato is largely regarded as a sweet crop in Bawku. This shows the existence of different consumer segments with different attribute preferences. In Nigeria, Dan China (also a low sweet variety) was also highly preferred over most sweet types.

**Table 4:** Consumer Liking Scores of all Cultivars Used for Both Market Surveys (A) in Ghana and Community Surveys in Both Ghana and Nigeria (B)

(A) Ghana markets only			(B) Ghana and Nigeria		
Regions	Genotype	Overall liking	Country	Genotype	Overall liking
Combined regions	SARI‐Nan	6.9 ± 2.2^a^	Ghana	Obare	6.8 ± 2.2^a^
	SARI‐Tiemeh	6.6 ± 2.2^ab^		Amuskwera	6.6 ± 2.1^ab^
	PGA14351‐4	6.6 ± 2.2^ab^		Kuffour	6.3 ± 2.0^ab^
	Okumkom	6.5 ± 2.3^ab^		Purupuru	5.7 ± 2.4^b^
	CRI‐Bohye	6.2 ± 2.2^b^	Nigeria	Dan Izala	7.9 ± 1.3^a^
Accra	SARI‐Nan	7.4 ± 1.9^a^		Dan China	7.9 ± 1.1^a^
	PGA14351‐4	7.3 ± 1.9^a^		Dan Barmawa	7.7 ± 1.9^ab^
	SARI‐Tiemeh	7.0 ± 2.1^ab^		Madagali	7.7 ± 1.2^ab^
	CRI‐Bohye	6.9 ± 2.1^ab^		Dan Silver	7.7 ± 1.2^ab^
	Okumkom	6.6 ± 2.4^b^		Aragbe	7.7 ± 0.9^ab^
				Alausa	7.7 ± 1.3^ab^
Akatsi	SARI‐Nan	7.5 ± 1.6^a^		Elege	7.5 ± 1.3^ab^
	PGA14351‐4	6.2 ± 2.2^ab^		Tomude	7.3 ± 2.0^ab^
	Okumkom	6.1 ± 2.3^ab^		Dan Bakalori	7.3 ± 1.2^ab^
	SARI‐Tiemeh	6.0 ± 2.5^ab^		Pakurumon	7.1 ± 1.6^ab^
	CRI‐Bohye	5.3 ± 2.2^b^		Mother's Delight	6.5 ± 2.2^b^
Bawku	SARI‐Nan	7.1 ± 1.9^a^			
	Okumkom	7.0 ± 1.8^a^			
	CRI‐Bohye	6.6 ± 1.8^ab^			
	SARI‐Tiemeh	6.6 ± 1.9^ab^			
	PGA14351‐4	6.2 ± 2.1^b^			
	Okumkom	5.9 ± 2.5^a^			
	SARI‐Tiemeh	5.9 ± 2.5^a^			
	PGA14351‐4	5.9 ± 2.5^a^			
Cape Coast	SARI‐Nan	5.5 ± 2.7^a^			
	CRI‐Bohye	4.9 ± 2.5^a^			

Means in column with similar letters are not significantly different (*P* < 0.05) (Hedonic Scale: 1 = Dislike extremely – 9 = Like extremely).

Consumers were generally grouped into three segments through agglomerative hierarchical clustering (AHC) of overall liking scores (Table 5 and Fig. 3) in Ghana. Segment 1, constituting about 37% of consumers, had SARI‐Nan and Okumkom (which vary greatly in appearance and texture attributes) as their most preferred cultivars with CRI‐Bohye and PGA14351‐4 least preferred. This group would be interested in sweet snacks since SARI‐Nan and Okumkom were described in DSA as highest in sweet taste, 6.7 and 5.3, respectively, while CRI‐Bohye and PGA14351‐4 were lowest in sweet taste with an average intensity score of only 2.5. Segment 2, made up of 24% of consumers, rated CRI‐Okumkom, PGA14351‐4 and SARI‐Tiemeh as their preferred cultivars. This group seemed to prefer high dry matter cultivars with fried product textural attributes of low sogginess and high dryness. Segment 3, with 39% of respondents, preferred orange‐fleshed cultivars (SARI‐Nan, PGA14351‐4 and CRI‐Bohye). Thus, in combination with segment 1, SARI‐Nan emerged as the most preferred cultivar in terms of overall acceptability in the study. Despite reports suggesting textural attributes are critical to fried product acceptability (Varela *et al*., 2008, Tortoe *et al*., 2014), orange‐flesh colour and its perceived associated nutritional value appeared to be additional driving forces in consumer acceptability (Tomlins *et al*., 2012; Sugri *et al*., 2012; Ekesa *et al*., 2017).

**Table 5:** Overall Liking Means for Each Cluster Derived from Consumer Market Survey in Ghana

Genotype	C1 (37%)	C2 (24%)	C3 (39%)
Nan	7.60 ± 1.4^a^	5.02 ± 2.6^c^	7.45 ± 1.2^a^
Okumkom	7.55 ± 1.1^a^	7.72 ± 1.7^a^	4.88 ± 2.3^c^
SARI‐Tiemeh	6.51 ± 2.0^b^	7.33 ± 1.6^ab^	6.15 ± 2.6^b^
PGA14351‐4	5.58 ± 2.3^c^	7.18 ± 1.7^ab^	7.08 ± 2.0^a^
Bohye	5.41 ± 2.1^c^	6.60 ± 2.2^b^	6.75 ± 2.1^ab^

Means in column with similar letters are not significantly different (*P* < 0.05) (Scale: 1 = Dislike extremely – 9 = Like extremely).

Regressing the various consumer segments onto the descriptive data highlighted the attributes driving consumer choices (Fig. 3). Consumer segment 1 was found to be driven by sweet taste, crispness, crunchiness, mealiness and fibrous appearance. Cluster decomposition (Table 6) showed that 34.7% of the people surveyed in Bawku (Northern Ghana), where sweetpotato production is highest among the selected regions, belong to this group. Familiarity of crop could have played a role since farmers in this region predominantly cultivate these sweet types. There were more consumers with lower levels of education in this group and consumers indicated preferences for moderately sweet (37.9%) and highly sweet (47.6%) cultivars. A preferred genotype could be an orange‐fleshed, sweet, high dry matter sweetpotato. This is why segment 1 had SARI‐Nan and CRI‐Okumkom as their most preferred cultivars. Segment 2 being the smallest group, was found to be driven by dry texture and yam flavour (Fig. 3). This group seemed to favour sweetpotatoes with characteristic yam attributes and could be an entry point for cultivars with very low or no sweetness. While 41.0% of the people in this group were less than 25 years, all the people above 65 years interviewed were also in this group. The chi‐square probability (*P *= 0.035) value indicates that age group distribution is dependent on type of cluster. Older people are more likely to prefer sweetpotatoes with characteristic attributes of this segment. The last group was driven by flavour attributes such as doughnut, ripe plantain and palm nutty flavours and uniform colour, which were all associated with orange‐fleshed cultivars. In terms of preference for orange‐fleshed cultivars, most consumers were in this group (47.8%). Younger consumers (ages below 25 years) were highest in this group. This was in line with the findings of Tomlins *et al*. (2007) that even younger children than those in this study are attracted by the orange colour. This group also contained people with higher levels of education, who may be more likely to be aware of the nutritional benefits of orange‐fleshed sweetpotatoes, influencing their decision regardless of other attributes. SARI‐Nan, PGA14351‐4 and CRI‐Bohye, all orange‐fleshed, were the most preferred cultivars for this group. Overall, sweetpotato can be said to be a seasonal crop and consumption is still low even during the peak season (occasional consumers = 32.3–44.8%) (Table 6). This may be due to low levels of nutritional knowledge (58.9–66.3%) among the general populace indicating that the higher preference for orange‐flesh (36.1–47.8) compared to other flesh colours could largely be due to their aesthetic appeal. As reported by Sugri *et al*. (2012), sweetpotatoes are considered a snack food (54.4%‐59.7%) and like most snacks, fries are preferred to boiled (62.7–73.4%) roots.

**Table 6:** Cluster Decomposition of Consumer Market Survey in Ghana

Question	Answers	C1 (*n *= 121)	C2 (*n *= 81)	C3 (*n *= 130)	Overall (*n *= 332)	Chi‐square probability
Location	Accra	30.6%	39.8%	45.5%	38.7%	0.092
	Akatsi	12.9%	3.6%	8.2%	8.8%	
	Bawku	34.7%	31.3%	26.1%	30.5%	
	Cape Coast	21.8%	25.3%	20.1%	22.0%	
Gender	Female	54.0%	62.7%	59.7%	58.4%	0.431
	Male	46.0%	37.3%	40.3%	41.6%	
Age group	18–24	47.6%	41.0%	53.7%	48.4%	
	25–34	26.6%	26.5%	20.9%	24.3%	0.035
	35–44	12.9%	13.3%	14.2%	13.5%	
	45–54	8.9%	8.4%	6.0%	7.6%	
	55–64	4.0%	4.8%	5.2%	4.7%	
	65–74	0.0%	6.0%	0.0%	1.5%	
Education	Illiterate	21.0%	21.7%	17.9%	19.9%	0.826
	JHS	29.0%	22.9%	23.1%	25.2%	
	SHS	21.8%	26.5%	28.4%	25.5%	
	Tertiary	28.2%	28.9%	30.6%	29.3%	
Ever eaten sweetpotato	Yes	97.6%	97.6%	97.0%	97.4%	0.950
	No	2.4%	2.4%	3.0%	2.6%	
Frequency of consumption during peak season	Never	.8%	1.2%	3.0%	1.8%	0.084
	Everyday	16.1%	9.6%	15.7%	14.4%	
	Thrice a week	15.3%	21.7%	14.9%	16.7%	
	Once a week	24.2%	18.1%	11.2%	17.6%	
	Once a month	11.3%	4.8%	10.4%	9.4%	
	Occasionally	32.3%	44.6%	44.8%	39.9%	
Frequency of consumption during lean season	Never	15.3%	22.9%	26.1%	21.4%	0.162
	Everyday	.8%	1.2%	3.0%	1.8%	
	Thrice a week	3.2%	7.2%	4.5%	4.7%	
	Once a week	20.2%	9.6%	10.4%	13.8%	
	Once a month	9.7%	4.8%	7.5%	7.6%	
	Occasionally	50.8%	54.2%	48.5%	50.4%	
Preferred form of consumption	Boiled	25.0%	36.1%	27.6%	28.7%	0.441
	Fried	73.4%	62.7%	70.9%	69.8%	
	Roasted	.8%	0.0%	1.5%	.9%	
	Raw					
	Processed product	.8%	1.2%	0.0%	.6%	
	Other					
Kind of food sweetpotato belong	Main meal	39.5%	43.4%	41.0%	41.1%	0.976
	Snack	59.7%	55.4%	58.2%	58.1%	
	Other	.8%	1.2%	.7%	.9%	
Where sweetpotato is obtained	Farm	12.9%	9.6%	11.2%	11.4%	0.932
	Market	83.9%	86.7%	85.8%	85.3%	
	Gift	1.6%	2.4%	.7%	1.5%	
	Other	1.6%	1.2%	2.2%	1.8%	
Preferred sweetness level	Non‐sweet	2.4%	4.8%	4.5%	3.8%	0.389
	Low sweet	12.1%	16.9%	20.9%	16.7%	
	Moderately sweet	37.9%	34.9%	30.6%	34.3%	
	High sweet	47.6%	42.2%	44.0%	44.9%	
Preferred flesh colour	White	43.5%	49.4%	41.0%	44.0%	0.534
	orange	45.2%	36.1%	47.8%	44.0%	
	Yellow	8.1%	13.3%	8.2%	9.4%	
	Purple	3.2%	1.2%	3.0%	2.6%	
Knowledge about nutritional benefit	Yes	41.1%	33.7%	40.3%	39.0%	0.522
	No	58.9%	66.3%	59.7%	61.0%	

C1: Consumer segment 1; C2: Consumer segment 2; C3: Consumer segment 3.

The aesthetic and nutritional value of beta‐carotene makes cultivars difficult to overlook regardless of other attributes and provides guidance to breeders on what attributes of the orange‐fleshed types need to be improved. Moreover, fried products can potentially serve as a medium for supplying vitamin A to vulnerable populations (Sulaeman *et al*, 2002) as the presence of oil enhances the bioaccessibility of the beta‐carotene for the human body (Tumuhimbise *et al*., 2009). Interestingly, while oiliness and sogginess could be a good quality attribute for consumers, especially children who like soft products and oily flavours, processors perceive it as an undesirable quality attribute (Ssali *et al*., 2020). Processors are profit driven and the greater amount of oil absorbed by these orange‐fleshed varieties raises their cost. This may contribute to low adoption of OFSP as a fried product unless breeders can develop improved OFSP with reduced oil absorption capacity, or processors adopt techniques such as parboiling, drying or coating to minimise oil absorption.

## Conclusion

Although sweetpotato cultivars possess diverse sensory attributes affecting frying quality and consumer preference, all cultivars in this study were acceptable across the locations regardless of the differences in sensory attributes. Acceptability of food is affected by many factors, which may be related to the individual, the food or the environment in which the food is consumed. Greater insights were obtained when trained sensory panels were used, combined with consumer market assessments of the same cultivars. Consumers generally preferred the deep orange‐fleshed and sweet cultivars (SARI‐Nan and Kuffour) compared to the less sweet type (PGA14351‐4) in Ghana. However, consumer segmentation showed that different cultivars were preferred by different consumer groups due to their unique attributes. Three consumer segments with varying attribute preferences were identified. One group preferred sweetpotatoes with sweet taste, crispy, crunchy and mealy textures, while another group was driven by dry texture and yam flavour. The third group was predominantly influenced by attractive colour, flavour and possibly softer textures. The test of independency showed that only age group was dependent on segment groupings. Community surveys in Ghana and Nigeria also indicated a similar trend. Though most currently available orange‐fleshed cultivars are generally perceived as poor candidates for fried products by processors due to higher oil consumption than other available cultivars, they command high consumer demand due to their attractive colour and unique flavour attributes. Sweetpotato cultivars of any colour, with dry, crispy, mealy texture and moderately sweet taste could be an ideal sweetpotato for many consumers. There is also a potential market for varieties with low sweetness with the above mentioned attributes. Clearly, these findings will contribute to the development of improved product profiles for sweetpotato breeders in West Africa. These findings could also aid industries to developed appropriate products to reach targeted consumers.

## Author contribution

**Eric Kuuna Dery:** Conceptualization (equal); Data curation (lead); Formal analysis (lead); Investigation (lead); Methodology (lead); Visualization (supporting); Writing‐original draft (lead); Writing‐review & editing (lead). **Edward E. Carey:** Conceptualization (supporting); Investigation (equal); Methodology (supporting); Supervision (equal); Writing‐original draft (supporting); Writing‐review & editing (equal). **Reuben Ssali:** Investigation (equal); Supervision (equal); Writing‐original draft (supporting); Writing‐review & editing (equal). **Jan W. Low:** Project administration (lead); Resources (lead); Supervision (supporting); Writing‐review & editing (equal). **Suzanne M Johanningsmeier:** Methodology (supporting); Validation (supporting); Writing‐original draft (equal); Writing‐review & editing (equal). **IBOK NSA ODURO:** Supervision (supporting); Writing‐original draft (supporting); Writing‐review & editing (supporting). **Abena Boakye:** Data curation (supporting); Formal analysis (supporting); Investigation (supporting); Supervision (supporting); Validation (supporting); Writing‐original draft (supporting). **Rachel M. Omodamiro:** Investigation (supporting). **Hauwa Ladi Yusuf:** Investigation (supporting); Writing‐review & editing (supporting).

## Conflict of interest

The authors declare no conflict of interest in this work.

## Ethical approval

Children were not used in the study. Respondents were informed about the study, they could stop the interview at any point, written consent from sensory panellists and from consumers participating in this study were obtained and the research respected the rules of voluntary participation and anonymity. Food samples were prepared according to good hygiene and local practices.

## Limitation of the work

A blindfold or red lightening system could have been used during the descriptive evaluation to mask the effect of colour on other attributes. Again, unlike the market survey and descriptive sensory analysis that used French fries sizes, the community survey employed chunk fries which were bigger.

## Supporting information


**Table S1.** Average scores and standard deviations of descriptive sensory characteristics for French fried sweetpotato.

## Data Availability

Data available upon request from the authors.

## Note

^1^ The dislike of anything unfamiliar or unknown.
