# Supplementary material for: Sensory characteristics and consumer segmentation of fried sweetpotato for expanded markets in Africa
Source: Int J Food Sci Technol. 2020 Nov 5;56(3):1419–31. doi: 10.1111/ijfs.14847 (PMC7984081; doi:10.1111/ijfs.14847)
Supplement: Supplementary file 1 — Table S1. Average scores and standard deviations of descriptive sensory characteristics for French fried sweetpotato. [file IJFS-56-1419-s001.docx]

**Supplementary Table 1. Average scores and standard deviations of descriptive sensory characteristics for French fried sweetpotato cultivars used for Ghana and Nigeria Community Survey**

|  |  |  | **Cultivars** |  |  |  |  |  |
| --- | --- | --- | --- | --- | --- | --- | --- | --- |
| **Attributes** | **AL** | **AR** | **DB** | **DBM** | **DC** | **DI** | **MA** | **EL** |
| **Colour UniformityA** | 8.1±1.1^ab^ | 8.1±0.9^a^ | 8.0±1.1^abc^ | 8.1±1.0^ab^ | 6.5±1.2^e^ | 8.0±1.2^abc^ | 7.0±1.00.9^cde^ | 7.4±0.9^abcde^ |
| **Surface BrowningA** | 1.2±1.0^fgh^ | 0.3±0.4^h^ | 1.7±0.9^efg^ | 0.8±1.0^gh^ | 2.2±0.9^bcdef^ | 3.5±1.4^ab^ | 1.9±1.2^defg^ | 4.7±1.4^a^ |
| **FibrousnessA** | 2.5±0.9^abc^ | 1.1±1.0^ef^ | 0.8±0.7^f^ | 0.8±0.9^f^ | 1.2±1.1^def^ | 0.6±0.9^f^ | 1.2±0.9^def^ | 1.5±1.2^cdef^ |
| **CrispnessTx** | 3.4±1.1^c^ | 1.0±0.9^de^ | 4.8±1.1^ab^ | 0.7±0.7^de^ | 0.6±0.8^de^ | 1.3±0.9^d^ | 0.5±0.6^de^ | 0.7±0.9^de^ |
| **SogginessTx** | 1.2±0.7^fg^ | 3.3±1.3^cd^ | 2.2±0.9^defg^ | 6.0±1.4^b^ | 6.3±2.0^b^ | 3.1±1.4^cde^ | 6.8±1.4^ab^ | 3.2±1.3^cd^ |
| **OilnessTx** | 1.9±0.9^defg^ | 3.6±1.2^bc^ | 1.1±0.6^g^ | 2.9±1.0^cd^ | 3.3±1.2^c^ | 2.7±1.2^cdef^ | 4.6±1.0^b^ | 3.1±1.2^c^ |
| **FibrousneTx** | 2.1±1.0^a^ | 1.0±0.7^cd^ | 0.7±0.7^cd^ | 0.6±0.8^cd^ | 0.5±0.5^cd^ | 0.5±0.8^cd^ | 0.4±0.8^cd^ | 0.4±0.4^d^ |
| **DrynessTx** | 5.7±1.5^bcd^ | 4.9±1.1^ab^ | 7.0±1.1^ab^ | 4.1±1.1^e^ | 5.0±1.3^cde^ | 4.6±1.1^de^ | 4.1±0.9^e^ | 4.9±1.0^cde^ |
| **CrunchinessTx** | 2.9±0.8^cd^ | 0.8±1.0^fg^ | 4.2±1.2^ab^ | 1.3±1.0^efg^ | 1.1±0.9^efg^ | 3.7±0.9^abc^ | 0.5±0.8^g^ | 1.0±1.1^fg^ |
| **MealinessTx** | 6.3±1.1^bcde^ | 6.7±1.1^abcd^ | 7.0±1.1^abc^ | 4.4±1.2^gh^ | 5.1±1.8^efgh^ | 6.1±1.0^cdef^ | 4.9±1.6^efgh^ | 5.7±1.1^defg^ |
| **SweetpotatoF** | 3.3±1.0^fg^ | 6.4±1.8^abc^ | 7.0±1.1^ab^ | 7.4±1.1^a^ | 5.9±1.3^bcd^ | 6.1±1.1^bc^ | 6.9±1.2^ab^ | 4.8±1.0^de^ |
| **YamF** | 7.1±1.2^c^ | 8.4±0.7^ab^ | 7.1±1.1^c^ | 4.9±1.1^d^ | 7.4±0.9^bc^ | 7.7±1.0^abc^ | 6.9±1.2^c^ | 5.3±1.0^d^ |
| **Roasted cocyamF** | 4.1±1.0^bcd^ | 3.6±1.2^cde^ | 3.1±1.4^def^ | 0.7±0.9^hi^ | 3.6±1.2^cde^ | 4.6±1.2^bc^ | 2.0±1.2^fgh^ | 4.7±1.0^bc^ |
| **Ripe plantainF** | 0.1±0.5^c^ | 0.5±0.8^c^ | 0.7±0.8^c^ | 0.9±0.9^c^ | 0.9±1.1^c^ | 0.4±0.8^c^ | 1.1±1.1^c^ | 2.8±1.2^b^ |
| **PalmnuttyF** | 0.1±0.4^d^ | 0.1±0.3^d^ | 0.1±0.4^d^ | 0.3±0.6^d^ | 0.1±0.4^d^ | 0.3±0.7^d^ | 0.2±0.4^d^ | 1.9±1.1^c^ |
| **DoughnutF** | 1.2±1.1^de^ | 2.7±0.9^bc^ | 2.7±0.9^ab^ | 4.4±1.3^ab^ | 1.8±1.1^cde^ | 3.3±1.4^ab^ | 2.7±1.6^c^ | 2.1±1.2^bcde^ |
| **SweetT** | 2.0±0.7^gh^ | 4.3±1.0^bc^ | 4.5±1.4^bc^ | 2.3±0.6^fgh^ | 1.4±0.8^h^ | 3.2±0.8^def^ | 2.4±0.9^efgh^ | 1.9±1.0^gh^ |

Means in row with similar letters are not statistically different (P>0.05). Intensities were generated using 0- to 9- scale with 0 signifying absence of a descriptor and 9 being highest intensity of a descriptor. Cultivars: AL=Alausa, AR=Aragbe, DB= Dan Bakolori, DBM= Dan Barmawa, DC=Dan China, DI=Dan Izala, MA= Madagali, EL=Elege. Descriptors ending: Ap= Appearance, Tx= Texture, F= Flavour, T= Taste

**Supplementary Table 1. Average scores and standard deviations of descriptive sensory characteristics for French fried sweetpotato cultivars used for Ghana and Nigeria Community Survey, cont.**

|  |  |  | **Cultivars** |  |  |  |  |  |
| --- | --- | --- | --- | --- | --- | --- | --- | --- |
| **Attributes** | **MD** | **PA** | **DS** | **TO** | **OB** | **KU** | **AM** | **PU** |
| **Colour UniformityA** | 7.7±0.7^abcd^ | 7.7±1.2^abcd^ | 6.5±1.1^e^ | 6.8±0.9^de^ | 6.4±0.2^e^ | 8.1±0.8^ab^ | 7.9±1.0^abcd^ | 7.1±0.8^abcde^ |
| **Surface BrowningA** | 0.9±1.2^fgh^ | 3.7±2.5^ab^ | 2.7±1.6^bcde^ | 3.2±1.2^bcd^ | 1.5±1.0^efgh^ | 2.1±1.2^cdefg^ | 0.8±1.0^gh^ | 3.2±1.1^bc^ |
| **FibrousnessA** | 1.2±0.9^cdef^ | 1.4±1.0^cdef^ | 2.2±1.2^bcd^ | 2.9±1.2^ab^ | 2.0±1.1^cde^ | 1.3±1.3^cdef^ | 0.7±0.8^f^ | 3.4±1.1^a^ |
| **CrispnessTx** | 0.4±0.6^de^ | 3.9±1.1^bc^ | 5.4±1.3^a^ | 2.7±2.0^c^ | 5.3±1.2^a^ | 3.4±1.5^c^ | 0.1±0.4^e^ | 5.1±1.6^ab^ |
| **SogginessTx** | 7.2±1.0^ab^ | 1.3±0.7^fg^ | 2.6±1.0^def^ | 2.0±1.4^defg^ | 1.8±1.1^efg^ | 4.1±1.2^c^ | 8.1±1.0^a^ | 1.2±0.9^g^ |
| **OilnessTx** | 4.7±1.2^b^ | 2.9±0.9^cde^ | 1.4±0.7^g^ | 1.6±0.9^fg^ | 1.8±0.9^efg^ | 3.1±1.0^cd^ | 6.8±0.9^a^ | 1.8±0.9^efg^ |
| **FibrousneTx** | 0.4±1.1^ab^ | 0.2±0.8^bcd^ | 0.2±0.7^cd^ | 0.2±0.8^bcd^ | 0.3±0.8^cd^ | 0.4±0.8^cd^ | 1.5±0.5^cd^ | 1.1±1.1^bc^ |
| **DrynessTx** | 0.8±0.6^g^ | 6.3±0.8^b^ | 6.5±1.1^b^ | 6.1±1.7^bc^ | 7.7±0.9^a^ | 4.4±1.2^e^ | 2.3±1.1^f^ | 8.0±0.9^a^ |
| **CrunchinessTx** | 4.6±0.7^a^ | 2.2±0.9^de^ | 3.43±1.0^bc^ | 2.9±1.2^cd^ | 1.6±1.1^ef^ | 3.3±1.1^bcd^ | 3.7±1.2^abc^ | 3.2±1.0^bcd^ |
| **MealinessTx** | 3.7±1.2^h^ | 4.9±1.8^efgh^ | 5.8±1.3^cdef^ | 6.6±0.9^abcd^ | 7.6±0.9^ab^ | 4.7±1.3^fgh^ | 4.3±1.2^h^ | 7.9±0.9^a^ |
| **SweetpotatoF** | 2.9±1.2^g^ | 6.2±1.1^abc^ | 4.5±0.9^ef^ | 6.3±1.4^abc^ | 6.1±1.2^bc^ | 5.4±1.0^cde^ | 6.8±0.9^ab^ | 6.8±0.9^ab^ |
| **YamF** | 0.1±0.4^f^ | 4.6±1.9^d^ | 5.5±0.9^d^ | 4.6±1.1^d^ | 8.7±0.6^a^ | 2.0±1.0^e^ | 5.1±1.2^d^ | 7.8±1.2^abc^ |
| **Roasted cocyamF** | 0.1±0.4^i^ | 4.1±1.6^bcd^ | 5.0±1.5^b^ | 2.6±1.1^defg^ | 4.7±1.2^bc^ | 2.4±1.6^efg^ | 1.3±1.4^ghi^ | 6.8±1.2^a^ |
| **Ripe plantainF** | 3.2±1.2^ab^ | 0.5±0.9^c^ | 0.5±0.8^c^ | 0.1±0.1^c^ | 0.4±0.1^c^ | 4.5±2.0^a^ | 3.3±1.2^ab^ | 0.4±0.7^c^ |
| **PalmnuttyF** | 3.1±0.8^b^ | 0.3±0.6^d^ | 0.1±0.3^d^ | 0.1±0.4^d^ | 0.1±0.3^d^ | 6.5±1.4^a^ | 3.1±0.9^b^ | 0.4±0.9^d^ |
| **DoughnutF** | 3.3±1.0^ab^ | 2.6±1.2^bcd^ | 2.7±1.2^bc^ | 1.1±1.1^e^ | 2.4±1.2^bcde^ | 2.1±1.1^bcde^ | 1.2±1.3^e^ | 1.9±1.2^cde^ |
| **SweetT** | 3.5±1.2^cde^ | 3.9±1.2^bcd^ | 2.3±0.9^fgh^ | 2.7±0.9^efg^ | 4.1±1.0^bcd^ | 4.5±1.0^bc^ | 5.7±1.2^a^ | 5.1±0.9^ab^ |

Means in row with similar letters are not statistically different (P>0.05). Intensities were generated using 0- to 9- scale with 0 signifying absence of a descriptor and 9 being highest intensity of a descriptor. Cultivars: MD= Mothers Delight, PA= Pakurumon, DS= Dan Silver, TO= Tomude, OB= Obare, KU=Kuffour, AM=Amusekwera, PU= Purupuru. Descriptors ending: Ap= Appearance, Tx= Texture, F= Flavour, T= Taste
